# How age and sex affect treatment outcomes for children with severe malnutrition: A multi‐country secondary data analysis

**DOI:** 10.1111/mcn.13596

**Published:** 2023-12-04

**Authors:** Susan Thurstans, Charles Opondo, Jeanette Bailey, Heather Stobaugh, Fabrizio Loddo, Stephanie V. Wrottesley, Andy Seal, Mark Myatt, André Briend, Michel Garenne, Andrew Mertens, Jonathan Wells, Rebecca Sear, Marko Kerac

**Affiliations:** ^1^ Department of Population Health London School of Hygiene and Tropical Medicine London UK; ^2^ Department of Medical Statistics London School of Hygiene and Tropical Medicine London UK; ^3^ National Perinatal Epidemiology Unit, Nuffield Department of Population Health University of Oxford Oxford UK; ^4^ International Rescue Committee New York USA; ^5^ Action Against Hunger New York USA; ^6^ Médecins Sans Frontières Paris France; ^7^ Emergency Nutrition Network Oxford UK; ^8^ UCL Institute for Global Health London UK; ^9^ Brixton Health, Llwyngwril Gwynedd UK; ^10^ Tampere Center for Child, Adolescent and Maternal Health Research Tampere University and Tampere University Hospital Tampere Finland; ^11^ Department of Nutrition, Exercise and Sports University of Copenhagen Copenhagen Denmark; ^12^ Institut de Recherche pour le Développement, UMI Résiliences Bondy France; ^13^ Department of Statistics and Population Studies University of the Western Cape Cape Town South Africa; ^14^ FERDI Université d'Auvergne Clermont‐Ferrand France; ^15^ MRC/Wits Rural Public Health and Health Transitions Research Unit, School of Public Health, Faculty of Health Sciences University of the Witwatersrand Johannesburg South Africa; ^16^ University of California, Berkeley School of Public Health Berkeley California USA; ^17^ Population, Policy and Practice Research and Teaching Department UCL Great Ormond Street Institute of Child Health London UK; ^18^ Maternal, Adolescent, Reproductive & Child Health Centre (MARCH) London School of Hygiene & Tropical Medicine London UK

**Keywords:** malnutrition, sex, treatment, undernutrition, wasting

## Abstract

Age and sex influence the risk of childhood wasting. We aimed to determine if wasting treatment outcomes differ by age and sex in children under 5 years, enroled in therapeutic and supplementary feeding programmes. Utilising data from stage 1 of the ComPAS trial, we used logistic regression to assess the association between age, sex and wasting treatment outcomes (recovery, death, default, non‐response, and transfer), modelling the likelihood of recovery versus all other outcomes. We used linear regression to calculate differences in mean length of stay (LOS) and mean daily weight gain by age and sex. Data from 6929 children from Kenya, Chad, Yemen and South Sudan was analysed. Girls in therapeutic feeding programmes were less likely to recover than boys (pooled odds ratio [OR]: 0.84, 95% confidence interval [CI]: 0.72–0.97, *p* = 0.018). This association was statistically significant in Chad (OR: 0.61, 95% CI: 0.39–0.95, *p* = 0.030) and Yemen (OR: 0.47, 95% CI: 0.27–0.81, *p* = 0.006), but not in Kenya and South Sudan. Multinomial analysis, however, showed no difference in recovery between sexes. There was no difference between sexes for LOS, but older children (24–59 months) had a shorter mean LOS than younger children (6–23 months). Mean daily weight gain was consistently lower in boys compared with girls. We found few differences in wasting treatment outcomes by sex and age. The results do not indicate a need to change current programme inclusion requirements or treatment protocols on the basis of sex or age, but future research in other settings should continue to investigate the aetiology of differences in recovery and implications for treatment protocols.

## Key messages

- There are few differences in recovery outcomes for wasting treatment by age and sex.
- Though differences are small, mean daily weight gain (g/kg/day) appears to be significantly lower in boys than girls. Likewise, though differences are small, younger children (6–23 months) often have a significantly longer mean length of stay compared with older children (24–59 months).
- The strength of our evidence does not indicate the need to change current inclusion criteria for wasting treatment programmes on the basis of age and sex but does suggest the need for further research to understand the effects of different confounders on treatment outcomes.

## 1 BACKGROUND

Undernutrition in all its forms remains a major contributor to child mortality. Child wasting, defined as weight‐for‐length or weight‐for‐height z‐score <−2 and/or mid‐upper‐arm circumference (MUAC) <125 mm, affects an estimated 49.5 million children under the age of five (GNR, 2022). Severe wasting (weight‐for‐length or weight‐for‐height z‐score <−3) is of particular concern since it is associated with a 12 times higher risk of mortality than experienced by well‐nourished children (Olofin et al., 2013). Renewed international attention to wasting recognises the need for accelerated progress towards effective integration of wasting treatment within strengthened health systems and improved efficiency of wasting treatment services (ENN, 2021; UNICEF, 2022).

Both age and sex influence the risk of wasting in childhood. In a recent meta‐analysis of 44 studies (S. Thurstans et al., 2020), we showed that boys are more likely to be wasted than girls (pooled odds ratio [OR]: 1.26, 95% confidence interval [CI]: 1.13–1.40). Other studies show similar findings; for example, a pooled analysis of 35 longitudinal cohorts (Mertens et al., 2020) from 15 low‐ and middle‐income countries (LMICs) showed male sex to be a predictor of wasting. Several studies exploring concurrent wasting and stunting both at population level and within wasting treatment programmes have also shown that overall, boys are more likely to be affected than girls (Imam et al., 2020; Isanaka et al., 2019; Khara et al., 2018; Myatt et al., 2018; Odei Obeng‐Amoako, Karamagi, et al., 2020; Odei Obeng‐Amoako, Myatt, et al., 2020; Odei Obeng‐Amoako, Wamani, et al., 2020). Sex differences are most likely caused by a complex interaction of social, environmental, physiological and genetic factors throughout the life cycle (S. Thurstans, 2022). Differences often begin in utero, particularly in conditions where maternal undernutrition is prevalent, and the impact of fetal growth restraint is often greater in males who are bigger than females at healthy z‐scores. Males also face a higher risk of infectious disease in infancy compared with females.

Previous studies have suggested that sex differences in undernutrition may be moderated by age (Costa et al., 2021; Myatt et al., 2018; S. Thurstans et al., 2020). The male disadvantage is greater among younger children, after which it disappears or, in some contexts, is reversed. Wasting has also been shown to peak in younger children between 0 and 3 months (Benjamin‐Chung et al., 2020; Mertens et al., 2023). Despite higher levels of wasting among children under 2 years compared with children aged 2–4 years (14% and 9%, respectively) (Karlsson et al., 2022), we recently demonstrated equivalent levels of associated mortality risk for younger (6–23 months) and older (24–59 months) wasted children and equivalent levels of mortality risk between wasted girls and boys (S. Thurstans et al., 2022).

The effects of age and sex on wasting treatment outcomes such as recovery, defaulting and non‐response have not, to our knowledge, been explored in depth and across multiple countries. This analysis was designed to fill that important evidence gap. Our aim is to determine whether age and/or sex influence treatment outcomes for children affected by wasting and, if associations are found, to discuss potential implications for policy and practice.

## 2 METHODS

### 2.1 Study design

This was a secondary analysis of multi‐country cohort data following STROBE guidelines (Vandenbroucke et al., 2007). We assessed whether there were differences in wasting treatment outcomes in children under 5 years by age and sex.

### 2.2 Study setting and participants

The data used for this analysis is from a multi‐country cohort compiled for ‘stage 1’ of the ComPAS study and is described elsewhere (Chase et al., 2020). In brief, the initial aim of this dataset was to help design a simplified MUAC‐based treatment protocol for children with acute malnutrition and to assess the theoretical performance of MUAC‐based delivery of a standard dose of ready‐to‐use therapeutic food (Chase et al., 2020). The data were collected from programmes providing standard treatment for wasting in four LMICs, three in Africa (Kenya, Chad and South Sudan) and one in Western Asia (Yemen). The data from South Sudan was collected by Médecins Sans Frontières (MSF)‐France in 2010. The International Rescue Committee collected data from Chad in 2013–2014, Kenya in 2012–2014 and Yemen in 2014 (Chase et al., 2020).

We focused on children aged 6–59 months in this dataset, stratified into younger (6–23 months) and older (24–59 months) age groups. Admission to either outpatient‐based therapeutic feeding programmes (TFP) or supplementary feeding programmes (SFP) was recorded using a unique child ID. Children enroled in this dataset were required to be clinically well. Each follow‐up visit was recorded using the same ID. Anthropometric measurements were recorded at each visit, weekly for severe wasting and every 2 weeks for moderate wasting. Treatment followed national protocols based on standard international criteria. Therapeutic rations for severe wasting were provided based on weight (200 kcal/kg/day). Supplementary rations for children with moderate wasting were provided as a standard ration which varied by country (Chase et al., 2020).

### 2.3 Variables

The dataset contained information on weight and height, the presence of oedema, MUAC, age, country, and which treatment programme children were enroled in, TFP for severe wasting, or SFP for moderate wasting. Our treatment outcomes of interest were recovery (TFP recovery criteria was MUAC ≥ 11.5 cm OR WFH/L ≥ −3z‐score for two consecutive weeks AND no bilateral pitting oedema, SFP recovery criteria was MUAC ≥ 12.5 cm OR WFH/L ≥ −2z‐score for two consecutive weeks); death, defined as a death occurring while enroled in the programme and assessed by verbal autopsy; default, defined as absence for two consecutive visits; and non‐response, defined as a child not responding to the treatment provided within 3 months (Kenya_Ministry_of_Health, 2009; Sudan, 2009, 2017; Yemen. Ministry of Public, Population. Yemen. Central Statistical, Pan Arab Programme for Family, & DHS, 2014). Transfers referred to either movement within different components of the programme or movements for further medical intervention. We were not able to determine individual reasons for transfers.

We also analysed length of stay (LOS), defined as the period of time between admission and discharge for those children who recovered, and daily weight gain g/kg/day, defined as: [*discharge weight* (*g*) *minus minimum weight* (*g*)]/[*minimum weight* (kg) × *the number of days between minimum weight and discharge day*] (MSF, 1995). Possible differences by sex and age were assessed for all outcomes. Children were stratified into two groups according to age at admission: 6–23 months and 24–59 months.

In addition to analysis by TFP and SFP enrolment, we performed the same treatment‐outcomes analysis and LOS and daily weight gain analyses on two further subgroups. The first was children with WAZ < −3, in light of the recent inclusion of WAZ as a means of identifying wasted children in recently revised WHO guidance for the management of acute malnutrition. The second subgroup was formed of children who were both wasted and stunted, in light of evidence demonstrating a higher prevalence of concurrent wasting and stunting in boys compared with girls. We defined this subgroup as all children with both baseline HAZ and WHZ score ≤−2.

We used the WHO classifications of severe wasting, defined as WLZ/WHZ < −3 or MUAC < 115 mm, and moderate wasting, defined as WLZ/WHZ between −3 and −2 z‐score or MUAC < 125 mm. We also included children with kwashiorkor in our analysis, defined as a child with bilateral pitting oedema.

### 2.4 Statistical methods

Statistical analysis was conducted using Stata V.16 (StataCorp 2017, Stata Statistical Software). Data was cleaned and excluded if age, sex or outcome variables were missing. Children under 6 months and over 5 years of age at admission to the programmes were also excluded from the analysis. Z‐scores were calculated using the 2006 WHO Child Growth Standards (WHO, 2006). We plotted data against normal distributions using quantile‐normal plots for each of the admission indices or criteria (WH/LZ, WAZ, H/LAZ<, MUAC and oedema). Values identified as implausible outliers were excluded (WHO, 2019).

For the treatment outcome variables (recovery, death, default, transfer and non‐response), we used logistic regression to calculate crude odds ratios and 95% CIs for the association between age and sex and the outcome indicators of interest. For each model, all other outcomes were used as the reference category modelling the likelihood of recovery, for example, recovery versus all other outcomes (death, default, transfer and non‐response). We further adjusted the analysis for baseline WH/LZ and HAZ, country, and age and sex. We also performed crude and adjusted multinomial analysis of outcomes by sex as a sensitivity test, using each of the different outcomes as the reference group.

For LOS and daily weight gain, we used linear regression to calculate differences in mean LOS and daily weight gain with 95% CIs for age and sex. Here, we also adjusted for baseline WH/LZ and HAZ, country, age and sex.

For all of the above analyses, we fit logistic regression models with interaction terms between age and sex and performed likelihood ratio (LR) tests between models with and without interactions to determine statistical significance of potential interactions.

### 2.5 Ethical approval

Ethical approval for stage 1 of the ComPAS trial was from the London School of Hygiene and Tropical Medicine Ethics Committee (reference number 11826). Further permission was granted for this analysis of the data by the same committee (reference number 26401).

## 3 RESULTS

### 3.1 Study characteristics

**Figure 1:**
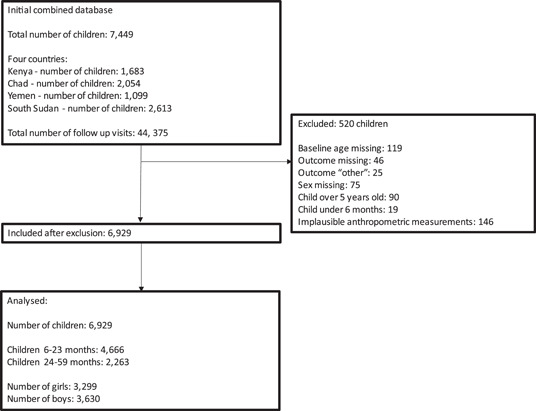
Flow diagram.

Figure 1 shows the study flow diagram. Data originated from four countries, Kenya, Chad, Yemen and South Sudan, and contained information from a total of 44,375 follow‐up visits for 7449 children. After data cleaning, 520 children were excluded from the analysis either due to missing data, not meeting inclusion criteria or implausible anthropometric measures. Following these exclusions, 6929 children were included in the analysis.

### 3.2 Children's nutrition status and treatment outcomes

Table 1 presents the baseline characteristics of the dataset. The data includes 3299 (47.6%) girls and 3630 (52.4%) boys. A total of 4666 (67.3%) were aged 6–23 months on admission and 2263 (32.7%) were aged 24–59 months on admission. The mean age at admission was 19.4 months (*SD* 12.5) for girls and 19.4 months (*SD* 12.5) for boys. For individual countries, the average age at admission was 21.3 months (*SD* 14.2) in Kenya, 16.6 months (*SD* 9.1) in Chad, 26.1 months (*SD* 16.7) in Yemen and 17.4 months (*SD* 9.6) in South Sudan.

**Table 1:** Baseline characteristics.

	6–23 Months		24–59 Months		
	Female	Male	Female	Male	Total
Country	*n* (%)	*n* (%)	*n* (%)	*n* (%)	*n* (%)
Kenya	495 (20.2)	433 (19.6)	355 (30.2)	293 (27.0)	1576 (22.8)
Chad	878 (35.8)	695 (31.3)	223 (19.0)	194 (17.8)	1990 (28.7)
South Sudan	767 (31.2)	856 (38.7)	333 (28.3)	361 (33.2)	2317 (15.1)
Yemen	314 (12.8)	228 (10.3)	265 (22.5)	239 (22.0)	1046 (33.4)
**Treatment site**					
TFP^a^	1327 (54.1)	1272 (57.5)	534 (45.4)	521 (47.9)	3654 (52.7)
SFP^b^	1127 (45.9)	940 (42.5)	642 (54.6)	566 (52.1)	3275 (47.3)
**Outcomes TFP**					
Recovered	928 (69.9)	870 (68.4)	365 (68.4)	361 (69.3)	2524 (69.1)
Death	1 (0.1)	3 (0.2)	0 (NA)	1 (0.2)	5 (0.1)
Default	240 (18.1)	259 (20.4)	118 (22.1)	118 (22.6)	735 (20.1)
Transfer	122 (9.2)	110 (8.6)	45 (8.4)	32 (6.1)	309 (8.5)
Non‐response	36 (2.7)	30 (2.4)	6 (1.1)	9 (1.7)	81 (2.2)
**Outcomes SFP**					
Recovered	832 (73.8)	700 (74.5)	431 (67.1)	370 (65.4)	2333 (71.2)
Death	3 (0.3)	1 (0.1)	3 (0.5)	2 (0.3)	9 (0.3)
Default	176 (15.6)	159 (16.9)	182 (28.3)	155 (27.4)	672 (20.5)
Transfer	102 (9.1)	62 (6.6)	19 (3.0)	27 (4.8)	210 (6.4)
Non‐response	14 (1.2)	18 (1.9)	7 (1.1)	12 (2.1)	51 (1.6)
**Totals**	**2454**	**2212**	**1176**	**1087**	**6929**
No cases oedema in TFP	7 (0.5)	8 (0.6)	16 (3.0)	18 (3.5)	49 (1.3)

Mean anthropometry TFP	Mean (*SD*)	Mean (*SD*)	Mean (*SD*)	Mean (*SD*)	Mean (*SD*)
Baseline WHZ	−3.27 (1.04)	−3.67 (0.94)	−3.54 (0.76)	−3.80 (0.90)	−3.53 (0.97)
Baseline WAZ	−3.27 (0.98)	−3.59 (0.90)	−3.77 (1.05)	−3.78 (1.04)	−3.53 (1.00)
Baseline HAZ	−1.62 (1.60)	−1.90 (1.67)	−2.36 (1.69)	−2.46 (1.78)	−1.95 (1.69)
Baseline MUAC mm	115.0 (8.28)	117.0 (8.28)	120.5 (9.36)	121.5 (9.16)	117.4 (8.92)
**Mean anthropometry SFP**					
Baseline WHZ	−1.78 (0.81)	−2.27 (0.86)	−2.02 (0.95)	−2.32 (0.94)	−2.06 (0.90)
Baseline WAZ	−2.31 (0.98)	−2.79 (0.95)	−2.65 (1.05)	−2.87 (0.98)	−2.61 (1.01)
Baseline HAZ	−1.75 (1.75)	−2.13 (1.86)	−2.12 (1.92)	−2.43 (1.75)	−2.05 (1.83)
Baseline MUAC mm	122.1 (4.64)	122.8 (5.21)	126.7 (6.27)	126.9 (6.54)	124.0 (5.90)
Daily weight gain TFP g/kg/day	5.14 (4.82)	5.22 (5.42)	5.79 (7.37)	5.39 (5.79)	5.30 (5.54)
Daily weight gain SFP g/kg/day	2.93 (3.53)	2.88 (4.03)	2.91 (4.41)	2.56 (3.20)	2.85 (3.81)
Length of stay TFP days	44.8 (28.4)	45.2 (29.3)	40.1 (32.9)	42.5 (30.1)	43.9 (29.7)
Length of stay SFP days	50.0 (29.7)	51.6 (32.8)	52.2 (33.2)	51.2 (34.0)	51.0 (32.0)

Abbreviations: SFP, supplementary feeding programmes; TFP, therapeutic feeding programmes.

^a^ Therapeutic feeding programme.

^b^ Supplementary feeding programme, percentages may not total 100 due to rounding.

Children entered either TFP (3654; 52.7%) or SFP (3275; 47.3%) at baseline. For TFP, the mean WHZ on admission was −3.53 (*SD* 0.97), the mean WAZ was −3.53 (*SD* 1.00), the mean HAZ was −1.95 (*SD* 1.69) and the mean MUAC was 117.4 mm (*SD* 8.92). There were 54 cases of oedema. For SFP, the mean WHZ on admission was −2.06 (*SD* 0.90), the mean WAZ was −2.61 (*SD* 1.01), the mean HAZ was −2.05 (*SD* 1.83) and the mean MUAC was 124.0 mm (*SD* 5.90). For both TFP and SFP, mean z‐scores were lower on average for boys compared with girls.

For TFP, the mean LOS was 43.9 days (*SD* 29.7) and the mean daily weight gain measured in g/kg/day was 5.30 (*SD* 5.54). For SFP, the mean LOS was 51.0 days (*SD* 32.0) and the mean daily weight gain measured in g/kg/day was 2.85 (*SD* 3.81).

Overall, 69.1% of children in TFP and 71.2% of children in SFP achieved recovery following wasting treatment, falling below the recommended 75% (Sphere Association, 2018). Deaths were rare in this sample and fell within the Sphere indicator of 3% for both TFP (0.1%) and SFP (0.3%). Defaulting rates were higher than recommended for both TFP (20.1%) and SFP (20.5%).

### 3.3 Outcomes by sex

Tables 2a and 2b show crude and adjusted odds ratios for recovery, death, default, transfer and non‐response by sex for TFP and SFP. In the crude analysis of TFP outcomes, girls were more likely to recover than boys, though this difference was not statistically significant. After adjusting for potential confounders (age, country, HAZ at baseline and WHZ at baseline), however, we found that girls were less likely to recover than boys (adjusted OR: 0.84, 95% CI: 0.72–0.97, *p* = 0.018). We further looked at the odds of recovery by sex for each country individually (see Table S1a). After adjusting for sex, age and baseline anthropometry (HAZ and WHZ), we found that for TFP there was no difference in the odds of recovery between girls and boys in Kenya and South Sudan (Kenya adjusted OR: 0.66, 95% CI: 0.39–1.13, *p* = 0.130, South Sudan adjusted OR: 0.96, 95% CI: 0.81–1.15, *p* = 0.703). In Chad and Yemen, girls were less likely to recover than boys (Chad adjusted OR: 0.61, 95% CI: 0.39–0.95, *p* = 0.030, Yemen, adjusted OR: 0.47, 95% CI: 0.27–0.81, *p* = 0.006). There was no difference in odds of recovery between girls and boys in SFP programmes in both the pooled and individual country analyses.

**Table 2a:** Association between treatment outcomes and age and sex within subgroups of therapeutic and supplementary feeding.

	TFP					SFP				
Outcome	No	OR (95% CI)	*p* value	Adjusted OR (95% CI)^a^	*p* value	No	OR (95% CI)	*p* value	Adjusted OR (95% CI)^a^	*p* value
**Recovery**										
Male	1231/1793	REF				1070/1506	REF		REF	
Female	1293/1861	1.04 (0.90–1.20)	0.591	0.84 (0.72–0.97)	0.018	1263/1769	1.02 (0.87–1.18)	0.827	0.93 (0.79–1.10)	0.408
6–23	1798/2599	REF		REF		1532/2067	REF		REF	
24–59	726/1055	0.98 (0.84–1.15)	0.829	1.16 (0.99–1.37)	0.07	801/1208	0.69 (0.59–0.80)	0.000	1.03 (0.87–1.23)	0.711
				Interaction^b^	0.717				Interaction^b^	0.252
**Death**										
Male	4/1793	REF		REF		3/1506	REF		REF	
Female	1/1861	0.24 (0.03–2.15)	0.203	0.37 (0.04–3.39)	0.376	6/1769	1.71 (0.43–6.83)	0.451	1.54 (0.37–6.35)	0.550
6–23	4/2599	REF		REF		4/2067	REF		REF	
24–59	1/1055	0.62 (0.07–5.51)	0.664	0.27 (0.02–2.85)	0.275	5/1208	2.14 (0.57–8.00)	0.256	2.27 (0.54–9.45)	0.260
				Interaction^b^	NA^c^				Interaction^b^	0.666
**Default**										
Male	377/1793	REF		REF		314/1506	REF		REF	
Female	358/1861	0.89 (0.76–1.05)	0.178	1.05 (0.88–1.24)	0.598	358/1769	0.96 (0.81–1.14)	0.665	1.00 (0.83–1.21)	0.986
6–23	499/2599	REF		REF		335/2067	REF		REF	
24–59	236/1055	1.21 (1.02–1.44)	0.030	1.08 (0.90–1.30)	0.415	227/1208	2.00 (1.68–2.38)	<0.0001	1.20 (0.99–1.46)	0.066
				Interaction^b^	0.571				Interaction^b^	0.577
**Transfer**										
Male	142/1793	REF		REF		89/1506	REF		REF	
Female	167/1861	1.15 (0.91–1.45)	0.253	1.34 (1.05–1.71)	0.017	121/1769	1.17 (0.88–1.55)	0.279	1.45 (1.07–1.96)	0.017
6–23	232/2599	REF		REF		164/2067	REF		REF	
24–59	77/1055	0.80 (0.61–1.05)	0.110	0.73 (0.55–0.97)	0.027	46/1208	0.46 (0.33–0.64)	0.000	0.43 (0.30–0.63)	0.000
				Interaction^b^	0.483				Interaction^b^	0.007
**Non‐response**										
Male	39/1793	REF		REF		30/1506	REF		REF	
Female	42/1861	1.04 (0.67–1.61)	0.867	1.30 (0.83–2.05)	0.255	21/1769	0.59 (0.34–1.04)	0.067	0.80 (0.42–1.52)	0.494
6–23	66/2599	REF		REF		32/2067	REF		REF	
24–59	15/1055	0.55 (0.31–0.97)	0.040	0.43 (0.24–0.77)	0.005	19/1208	1.02 (0.57–1.80)	0.956	1.22 (0.65–2.29)	0.527
				Interaction^b^	0.372				Interaction^b^	0.707

*Note*: This table represents results from 5 sets of logistic regression models; ORs represent the likelihood of each outcome compared with all other outcomes.

Abbreviations: CI, confidence interval; OR, odds ratio.

^a^ Adjusted for sex, age, country, HAZ at baseline and WHZ at baseline.

^b^ Test for interaction between age group and sex.

^c^ No events, so test not comparable.

**Table 2b:** Association between treatment outcomes and age and sex within subgroups of children who are wasted and stunted (WaSt) and children with WAZ < −3.

	WAZ < −3					WaSt				
Outcome	No	OR (95% CI)	*p* value	Adjusted OR (95% CI)^a^	*p* value	No	OR (95% CI)	*p* value	Adjusted OR (95% CI)^a^	*p* value
**Recovery**										
Male	1425/2081	REF		REF		1073/1517	REF		REF	
Female	1218/1731	1.03 (0.93–1.14)	0.546	0.91 (0.82–1.01)	0.085	811/1160	0.96 (0.81–1.14)	0.646	0.83 (0.70–1.00)	0.045
6–23	1735/2467	REF		REF		1171/1630	REF		REF	
24–59	908/1345	0.88 (0.76–1.01)	0.071	1.03 (0.92–1.16)	0.623	713/1047	0.84 (0.71–0.99)	0.039	1.08 (0.90–1.30)	0.387
				Interaction^b^	0.522				Interaction^b^	0.902
**Death**										
Male	4/2081	REF		REF		3/1517	REF		REF	
Female	2/1731	0.60 (0.11–3.28)	0.556	0.73 (0.13–4.13)	0.719	1/1160	0.44 (0.05–4.19)	0.472	0.63 (0.62–6.51)	0.701
6–23	3/2467	REF		REF		3/1630	REF		REF	
24–59	3/1345	1.84 (0.37–9.11)	0.457	0.85 (0.16–4.61)	0.852	1/1047	0.52 (0.05–4.99)	0.570	0.21 (0.02–2.73)	0.231
				Interaction^b^	0.751				Interaction^b^	NA^c^
**Default**										
Male	411/2081	REF		REF		285/1517	REF		REF	
Female	310/1731	0.89 (0.75–1.04)	0.148	0.95 (0.80–1.13)	0.552	201/1160	0.91 (0.74–1.11)	0.332	0.97 (0.79–1.20)	0.801
6–23	410/2467	REF		REF		245/1630	REF		REF	
24–59	311/1345	1.51 (1.28–1.78)	<0.001	1.28 (1.07–1.53)	0.007	241/1047	1.69 (1.39–2.06)	0.000	1.19 (0.96–1.48)	0.103
				Interaction^b^					Interaction^b^	
**Transfer**										
Male	190/2081	REF		REF		119/1517	REF		REF	
Female	160/1731	1.01 (0.81–1.26)	0.904	1.12 (0.89–1.40)	0.321	110/1160	1.23 (0.94–1.61)	0.134	1.40 (1.06–1.85)	0.018
6–23	247/2467	REF		REF		157/1630	REF		REF	
24–59	103/1345	0.75 (0.59–0.95)	0.016	0.74 (0.58–0.96)	0.022	72/1047	0.69 (0.52–0.93)	0.013	0.71 (0.52–0.96)	0.026
				Interaction^b^	0.727				Interaction^b^	0.768
**Non‐response**										
Male	51/2081	REF		REF		37/1517	REF		REF	
Female	41/1731	0.96 (0.64–1.46)	0.869	1.18 (0.77–1.81)	0.439	37/1160	1.32 (0.83–2.09)	0.242	1.51 (0.94–2.41)	0.087
6–23	72/2467	REF		REF		54/1630	REF		REF	
24–59	20/1345	0.50 (0.30–0.83)	0.007	0.46 (0.28–0.78)	0.004	20/1047	0.57 (0.34–0.96)	0.033	0.56 (0.33–0.97)	0.038
				Interaction^b^	0.647				Interaction^b^	0.485

*Note*: This table represents results from 5 sets of logistic regression models; ORs represent the likelihood of each outcome compared with all other outcomes.

Abbreviations: CI, confidence interval; OR, odds ratio.

^a^ Adjusted for sex, age, country, HAZ at baseline and WHZ at baseline.

^b^ Test for interaction between age group and sex.

^c^ No events, so test not comparable.

Girls were more likely than boys to be transferred out of the programme in the adjusted analysis for both TFP and SFP (adjusted OR: 1.34, 95% CI: 1.05–1.71, *p* = 0.017 and adjusted OR: 1.45, 95% CI: 1.07–1.96, *p* = 0.017, respectively).

We did not observe any differences between boys and girls in the odds of death, defaulting or non‐response in TFP or SFP.

We performed a multinomial sensitivity analysis to further explore outcomes by sex (see Table S2). Using recovery as the reference group, there was no statistically significant difference in the risk of death, default or non‐response compared with recovery between girls and boys. In the adjusted analysis, girls were more likely to be transferred out of TFP than to recover compared with boys (adjusted relative risk: 1.41, 95% CI: 1.10–1.80, *p* = 0.007). Using default or death as the reference group, there was no statistically significant difference in outcomes between girls and boys.

Table 3 shows mean crude and adjusted differences in LOS and daily weight gain by sex. For LOS, we did not observe any difference between girls and boys for either TFP or SFP. Girls had a higher mean daily weight gain than boys in TFP and SFP (mean adjusted difference: 0.61 g/kg/day, 95% CI: 0.24–1.04, *p* = 0.002 and mean adjusted difference: 0.30 g/kg/day, 95% CI: 0.00–0.61, *p* = 0.049, respectively).

**Table 3:** Mean differences in length of stay (LOS) and daily weight gain by age and sex within subgroups for TFP, SFP and children who are wasted and stunted or have WAZ < −3.

Outcome	Mean (*SE*)	Crude difference (95% CI)	*p* value	Adjusted difference (95% CI)^a^	*p* value
LOS TFP, mean (*SE*) days^b^					
Male	49.8 (0.81)	REF		REF	
Female	48.6 (0.81)	−1.25 (−3.50 to 1.00)	0.277	0.79 (−1.45 to 3.05)	0.486
6–23	51.0 (0.65)	REF		REF	
24–59	44.7 (1.18)	−6.28 (−8.75 to −3.80)	<0.001	−7.05 (−9.55 to −4.55)	<0.001
				Interaction	
LOS SFP, mean (*SE*) days^b^					
Male	54.1 (0.99)	REF		REF	
Female	52.9 (0.79)	−1.22 (−3.68 to 1.23)	0.329	0.33 (−2.17 to 2.83)	0.796
6–23	53.0 (0.75)	REF		REF	
24–59	54.3 (1.11)	1.26 (−1.31 to 3.84)	0.336	−5.25 (−7.94 to −2.56)	<0.001
				Interaction	0.642
LOS WaSt, mean (*SE*) days^b^					
Male	49.6 (0.86)	REF		REF	
Female	49.7 (0.86)	0.16 (−2.26 to 2.58)	0.897	1.96 (−4.48 to 4.40)	0.115
6–23	50.4 (0.77)	REF		REF	
24–59	48.4 (1.01)	−2.04 (−4.52 to 0.43)	0.106	−3.53 (−6.07 to −0.99)	0.007
				Interaction	0.778
LOS WAZ < −3, mean (*SE*) days^b^					
Male	51.1 (0.76)	REF		REF	
Female	49.9 (0.80)	−1.26 (−3.43 to 0.91)	0.255	0.24 (−1.97 to 2.45)	0.833
6–23	51.7 (0.66)	REF		REF	
24–59	48.3 (0.99)	−3.33 (−5.61 to −1.05)	0.004	−4.88 (−7.24 to −2.52)	<0.001
				Interaction	0.811
Daily weight gain, TFP, mean (*SE*) g/kg/day					
Male	5.27 (0.14)	REF		REF	
Female	5.33 (0.15)	0.05 (−0.34 to 0.46)	0.789	0.61 (0.24 to 1.04)	0.002
6–23	5.18 (0.11)	REF		REF	
24–59	5.59 (0.22)	0.41 (−0.03 to 0.86)	0.071	−0.03 (−0.47 to 0.41)	0.894
				Interaction	0.575
Daily weight gain, SFP, mean (*SE*) g/kg/day					
Male	2.77 (0.11)	REF		REF	
Female	2.92 (0.10)	0.15 (−0.14 to 0.44)	0.307	0.30 (0.00 to 0.61)	0.049
6–23	2.91 (0.09)	REF		REF	
24–59	2.74 (0.13)	−0.16 (−0.46 to 0.14)	0.288	−0.13 (−0.45 to 0.20)	0.432
				Interaction	0.404
Daily weight gain, WaSt, mean (*SE*) g/kg/day					
Male	4.31 (0.14)	REF		REF	
Female	4.67 (0.16)	0.36 (−0.06 to 0.77)	0.091	0.66 (0.26 to 1.07)	<0.001
6–23	4.38 (0.14)	REF		REF	
24–59	4.60 (0.16)	0.22 (−0.20 to 0.65)	0.302	−0.16 (−0.58 to 0.26)	0.444
				Interaction	0.566
Daily weight gain, WAZ < −3, mean (*SE*) g/kg/day					
Male	4.65 (0.14)	REF		REF	
Female	5.00 (0.15	0.35 (−0.05 to 0.75)	0.089	0.69 (0.30 to 1.08)	0.001
6–23	4.81 (0.12)	REF		REF	
24–59	4.82 (0.19)	0.01 (−0.41 to 0.43)	0.962	−0.09 (−0.51 to 0.33)	0.671
				Interaction	0.596

a Adjusted for sex, age, country, HAZ at baseline and WHZ at baseline.

b Measured for recovered children only.

### 3.4 Outcomes by age

Table 2a shows crude and adjusted odds ratios for recovery, death, default, transfer and non‐response by age for TFP and SFP. We found no differences in odds of recovery, death or default between the two age groupings. Older children had a lower risk of non‐response to treatment compared with younger children (OR: 0.43, 95% CI: 0.24–0.77, *p* = 0.005). In the crude analysis, older children attending SFP were more likely to default compared with the younger age group (OR: 2.00, 95% CI: 1.68–2.38, *p* < 0.0001); however, this was no longer the case after adjustment (adjusted OR: 1.20, 95% CI: 0.99–1.46, *p* = 0.066).

Older children were less likely to be transferred out of the programme compared with younger children in both TFP and SFP (adjusted OR: 0.73, 95% CI: 0.55–0.97, *p* = 0.027 and adjusted OR: 0.43, 95% CI: 0.30–0.63, *p* < 0.0001, respectively).

Table 3 shows mean differences in LOS and daily weight gain by age. Older children in TFP and SFP had a significantly shorter LOS than younger children (adjusted mean difference: −7.05 days, 95% CI: −9.55 to −4.55, *p* ≤ 0.0001 and −5.25 days, 95% CI: −7.94 to −2.56, *p* < 0.0001, respectively). For daily weight gain, we did not observe differences between age groups in either TFP or SFP.

### 3.5 Children with WAZ < −3

Table 2b shows crude and adjusted odds ratios for recovery, death, default, transfer and non‐response by age and sex for children with a WAZ < −3 at baseline. We found no difference between girls and boys for all outcomes. For age however, in both crude and adjusted analysis, older children were more likely to default (adjusted OR: 1.28, 95% CI: 1.07–1.53, *p* = 0.007), less likely to be transferred out of a programme (adjusted OR: 0.74, 95% CI: 0.58–0.96, *p* = 0.022), and had a lower risk of non‐response to treatment, compared with younger children (adjusted OR: 0.46, 95% CI: 0.28–0.78, *p* = 0.004).

Table 3 shows mean differences in LOS and daily weight gain by age and sex for children with WAZ < −3 at baseline. There was no difference between girls and boys for LOS, but after adjusting for potential confounders, older children had a shorter LOS compared with younger children (adjusted difference OR: −4.88 days, 95% CI: −7.24 to −2.52, *p* < 0.001). As with TFP and SFP, girls had a higher mean daily weight gain compared with boys (adjusted difference: 0.69 g/kg/day, 95% CI: 0.30–1.08, *p* = 0.001).

### 3.6 Children with concurrent wasting and stunting (WaSt)

Table 2b shows crude and adjusted odds ratios for recovery, death, default, transfer and non‐response by age and sex for children who were both wasted and stunted. Girls were less likely to recover than boys in both the crude and adjusted analysis (adjusted OR: 0.83, 95% CI: 0.70–1.00, *p* = 0.045). Girls were more likely to be transferred out of a programme than boys after adjusting for potential confounders (OR: 1.40, 95% CI: 1.06–1.85, *p* = 0.018). We found no difference between girls and boys for death, default and non‐recovery.

Table 3 shows mean differences in LOS and daily weight gain by age and sex for children who were wasted and stunted. We found no difference between girls and boys for LOS. For daily weight gain, boys had a lower mean gain compared with girls (adjusted mean difference: 0.66 g/kg/day, 95% CI: 0.26–1.07, *p* ≤ 0.001).

In terms of age (see Table 2b), as with TFP and SFP, we found that older children were less likely to be transferred out of a programme compared with younger children (adjusted OR: 0.71, 95% CI: 0.52–0.96, *p* = 0.026). We also found that older children had a lower risk of non‐response to treatment compared with younger children in both crude and adjusted analysis (adjusted OR: 0.56, 95% CI: 0.33–0.97, *p* = 0.038). Finally, older children had a shorter LOS than younger children (adjusted difference: −3.53 days, 95% CI: −6.07 to −0.99, *p* = 0.007) (see Table 3).

### 3.7 Interactions between age and sex

We sought to conduct subgroup analysis for the above tests to test for interaction between age and sex using LR tests. For two of the models, this was not possible (death and sex in TFP, death and sex for WaSt) due to too few events in the subgroups. We observed an interaction between age and sex in transfers out of SFP (*p* = 0.007). For all other models, we found no evidence that associations between treatment outcomes, LOS and daily weight gain varied by sex or age (see Tables 2a, 2b, 3).

## 4 DISCUSSION

We explored the impact of age and sex on outcomes following treatment for wasting. Overall, our findings show few differences in treatment outcomes between girls and boys and between age groups. Based on this evidence from these settings, this suggests no need to change current programme inclusion requirements or treatment protocols on the basis of sex or age.

Our findings showed that girls in TFP have 16% lower adjusted odds of recovery than boys (OR: 0.84, 95% CI: 0.72–0.97, *p* = 0.018). Individual country analysis showed the same association to be statistically significant in Chad and Yemen, but not in Kenya or South Sudan. Girls who were both wasted and stunted were also less likely to recover compared with boys (OR: 0.83, 95% CI: 0.70–1.00, *p* = 0.045). Baseline anthropometry appeared to be the main confounding variable. There was no difference between boys and girls in recovery outcomes for SFP or in our subgroup analysis of children with WAZ < −3. We were unable to adjust for social, economic, care and feeding practices and co‐morbidities, leaving the possibility of residual confounding. While changes in the magnitude of odds ratios after adjustment may indicate confounding, such changes can also occur in the absence of confounding due to the non‐collapsibility property of odds ratios (Greenland, 2021a, 2021b). Further multinomial analysis, conducted as a sensitivity test, demonstrated no statistical difference between recovery by sex in TFP. The finding that girls in TFPs are less likely to recover than boys is not therefore generalisable to all settings and should be interpreted with caution. Further research is needed to understand the effect of admission and discharge criteria, baseline anthropometry and other potential confounding factors such as social, health and care indicators.

We did not observe any differences in age or sex in relation to mortality. While this sample likely lacked sufficient power for this outcome, the finding is consistent with our recent meta‐analysis (S. Thurstans et al., 2022), showing no difference in the risk of mortality associated with wasting between boys and girls and between children under 2 years versus those 2–5 years. This highlights the importance of access to treatment for all children under 5 years, regardless of age and sex.

We observed lower mean weight gain in boys compared with girls in TFP, SFP, and WaSt and WAZ < −3 subgroups. Though differences are small, they might be explained by differences between girls and boys in lean and fat mass from birth onwards. Differences in body composition in infancy and early childhood have been documented whereby although girls are lighter at birth and during infancy, girls on average have less lean mass and more fat mass than males. This might then shape sex differences in weight gain (Andersen, 2013; Rodríguez, 2004). Future research into body composition and weight gain in wasting recovery and links to future health are needed. There might also be differences in the way that girls and boys respond to treatment. A recent meta‐analysis of SQ‐LNS supplementation demonstrated better growth in girls compared with boys in response to SQ‐LNS supplementation (Dewey et al., 2021). The authors suggest that this likely reflects greater potential in girls to respond to nutritional supplementation and an effect of early vulnerability in boys to adverse conditions, which might constrain responses to nutrition interventions.

We observed that girls are transferred out of programmes more often than boys for both SFP and TFP. This was also the case in our subgroup analysis of children who were both wasted and stunted. Younger children were consistently more likely to be transferred out of a programme than older children. It is difficult to speculate as to the reasons for this pattern as the data does not distinguish between transfers to other components of programmes such as inpatient, TFP or SFP, transfers to other sites, or medical transfers. Further research is needed to better understand this. We observed longer LOS and a higher risk of non‐response for younger children in TFP. We also observed a higher risk on non‐response for younger children in our subgroup analyses of children with concurrent wasting and stunting and children with WAZ < −3. It is again hard to speculate as to why without understanding factors such as which discharge criteria were used for each individual child and the presence or absence of co‐morbidities.

### 4.1 Limitations and recommendations for future research

The strength of this analysis lies in data originating from four different countries with large numbers of children. Almost all children were wasted, which enhances the validity of pooling the data and avoids the many complexities of analysing data on oedematous severe malnutrition (especially when it comes to weight‐based measures) (Frison et al., 2015). However, we also acknowledge limitations, many of which arise from the nature of the dataset. This data came from multiple locations and time periods and was collected by different non‐governmental organisations. As this data does not originate from carefully controlled research programmes, there are gaps in the information needed to draw further conclusions about the findings. For example, we did not have data to say exactly how children were treated; that is, in SFP, did a child receive ready‐to‐use supplementary food or fortified flours, or did children switch to RUSF or fortified flours once they reached a certain anthropometric threshold. Similarly, we did not have information on precise details of entry and exit criteria for each child, that is, admitted on the basis of low MUAC or low WHZ. The absence of children less than 6 months is a limitation to fully understanding sex differences in treatment outcomes, especially given that male vulnerability is often more pronounced in infancy. Children were required to be clinically well to be enroled in this dataset so there may be a degree of survivor or selection bias introduced as children admitted for inpatient care with the most severe presentations of wasting were not included. There may also be a possibility of survivor bias in the sample as this was not a community sample, but programme data. This sample also contained a higher number of females in three of the sites, with the exception of South Sudan. This is inconsistent with other population‐based figures showing higher risk of undernutrition in males (S. Thurstans et al., 2020).

The high levels of transfers and defaulters also introduce a risk of selection bias and highlight both the challenges in cohort data and the importance of ensuring allocated funding with programmes to follow‐up and fully understand reasons for transfers and defaults to ensure programme quality and accurate representation of performance indicators.

Crucially, the dataset did not contain information on potential confounders such as indicators of maternal education, care and feeding indicators, socio‐economic status and the presence or absence of co‐morbidities. This information would not only enable better understanding of the differences that were observed in this analysis but would also enable better understanding of the possible mechanisms underlying any differences and whether addressing behaviours would impact these differences. Future research should explore such factors including whether different admission and discharge criteria, different programmes in different geographical locations, and other potential confounders (e.g., social, economic and environmental factors, all of which impact outcomes from malnutrition) might produce different results. Research should also further explore the differences we identified in LOS and weight gain between younger and older children and girls and boys to better understand the different causes of growth failure, such as nutritional intake and feeding behaviours by age and sex and to determine if different treatment or prevention strategies are needed.

## 5 CONCLUSION

These findings show few differences in wasting treatment outcomes between girls and boys and between age groups. The results do not indicate any immediate case for a change in current programme inclusion requirements or treatment protocols on the basis of sex or age. Further research should use more formal study designs and more robust methods to investigate the aetiology of any sex or age differences in recovery and implications for treatment protocols.

## AUTHOR CONTRIBUTIONS

Susan Thurstans was responsible for the concept and design of the study, as well as the sourcing of data. Data was contributed by the International Rescue Committee and medicines sans Frontiers. Jeanette Bailey and Fabrizio Loddo supported in developing the data sharing agreement. Susan Thurstans led in the analysis of data with support from Charles Opondo. Susan Thurstans wrote the manuscript with contributions from all authors. All authors have read and approved the final manuscript.

## CONFLICT OF INTEREST STATEMENT

The authors declare no conflicts of interest.

## Supporting information

Supporting information.

## Data Availability

The data that support the findings of this study are available from the corresponding author upon reasonable request.
