# Supplementary material for: How age and sex affect treatment outcomes for children with severe malnutrition: A multi‐country secondary data analysis
Source: Matern Child Nutr. 2023 Dec 4;20(3):e13596. doi: 10.1111/mcn.13596 (PMC11168354; doi:10.1111/mcn.13596)
Supplement: Supplementary file 1 — Supporting information. [file MCN-20-e13596-s001.docx]

Supplementary table 1a. Association between recovery and age and sex within subgroups of TFP and SFP by individual country.

|  | TFP |  |  |  |  | SFP |  |  |  |  |
| --- | --- | --- | --- | --- | --- | --- | --- | --- | --- | --- |
| Country | No | OR (95% CI) | p value | Adjusted OR (95% CI)* | p value | No | OR (95% CI) | p value | Adjusted OR (95% CI)* | p value |
| Kenya |  |  |  |  |  |  |  |  |  |  |
| Male | 150/184 | REF |  | REF |  | 359/542 | REF |  | REF |  |
| Female | 173/219 | 0.88 (0.53-1.44) | 0.602 | 0.66 (0.39-1.13) | 0.130 | 397/631 | 0.86 (0.68-1.10) | 0.236 | 0.81 (0.64-1.05) | 0.109 |
|  |  |  |  |  |  |  |  |  |  |  |
| 6-23 | 229/304 | REF |  | REF |  | 413/624 | REF |  | REF |  |
| 24-59 | 95/99 | 7.78 (2.77-21.87) | <0.001 | 10.03 (3.45-29.15) | <0.001 | 343/549 | 0.85 (0.67-1.08) | 0.186 | 0.91 (0.70-1.18) | 0.476 |
| Chad |  |  |  |  |  |  |  |  |  |  |
| Male | 227/270 | REF |  | REF |  | 521/619 | REF |  | REF |  |
| Female | 285/360 | 0.72 (0.48-1.09) | 0.119 | 0.61 (0.39-0.95) | 0.030 | 637/741 | 1.15 (0.85-1.55) | 0.354 | 0.69 (0.47-1.00) | 0.053 |
|  |  |  |  |  |  |  |  |  |  |  |
| 6-23 | 401/491 | REF |  | REF |  | 916/1082 | REF |  | REF |  |
| 24-59 | 111/139 | 0.89 (0.55-1.43) | 0.629 | 1.00 (0.61-1.64) | 0.997 | 242/278 | 1.22 (0.83-1.79) | 0.318 | 1.61 (1.07-2.42) | 0.022 |
| Yemen |  |  |  |  |  |  |  |  |  |  |
| Male | 88/122 | REF |  | REF |  | 190/345 | REF |  | REF |  |
| Female | 112/182 | 0.62 (0.38-1.01) | 0.057 | 0.47 (0.27-0.81) | 0.006 | 229/397 | 1.11 (0.83-1.49) | 0.475 | 1.38 (1.00-1.91) | 0.052 |
|  |  |  |  |  |  |  |  |  |  |  |
| 6-23 | 117/181 | REF |  | REF |  | 203/361 | REF |  | REF |  |
| 24-59 | 83/123 | 1.14 (0.70-1.84) | 0.609 | 1.15 (0.70-1.92) | 0.573 | 216/381 | 1.02 (0.76-1.36) | 0.899 | 0.90 (0.66-1.22) | 0.498 |
| South Sudan |  |  |  |  |  |  |  |  |  |  |
| Male | 766/1217 | REF |  | REF |  | No SFP programme | | | | |
| Female | 722/1100 | 1.12 (0.95-1.33) | 0.177 | 0.96 (0.81-1.15) | 0.703 |  |  |  |  |  |
|  |  |  |  |  |  |  |  |  |  |  |
| 6-23 | 1051/1623 | REF |  | REF |  |  |  |  |  |  |
| 24-59 | 437/694 | 0.93 (0.77-1.11) | 0.411 | 0.90 (0.74-1.11) | 0.325 |  |  |  |  |  |
|  |  |  |  |  |  |  |  |  |  |  |

This table represents results from 5 sets of logistic regression models; ORs represent the likelihood of recovery compared with all other outcomes for each country

*adjusted for sex, age, HAZ at baseline and WHZ at baseline.

Supplementary table 1b. Association between recovery and age and sex within subgroups of children who are wasted and stunted (WaSt) or have WAZ <-3 by individual country.

|  | WaSt |  |  |  |  | WAZ <-3 |  |  |  |  |
| --- | --- | --- | --- | --- | --- | --- | --- | --- | --- | --- |
| Country | No | OR (95% CI) | p value | Adjusted OR (95% CI)* | p value | No | OR (95% CI) | p value | Adjusted OR (95% CI)* | p value |
| Kenya |  |  |  |  |  |  |  |  |  |  |
| Male | 102/150 | REF |  | REF |  | 177/258 | REF |  | REF |  |
| Female | 68/114 | 0.70 (0.42-1.56) | 0.161 | 0.65 (0.38-1.09) | 0.103 | 140/212 | 0.89 (0.60-1.31) | 0.555 | 0.83 (0.56-1.25) | 0.375 |
|  |  |  |  |  |  |  |  |  |  |  |
| 6-23 | 89/113 | REF |  | REF |  | 158/231 | REF |  | REF |  |
| 24-59 | 81/131 | 0.80 (0.48-1.33) | 0.389 | 0.79 (0.47-1.34) | 0.390 | 159/239 | 0.92 (0.62-1.35) | 0.665 | 0.99 (0.67-1.48) | 0.375 |
| Chad |  |  |  |  |  |  |  |  |  |  |
| Male | 521/628 | REF |  | REF |  | 548/668 | REF |  | REF |  |
| Female | 398/498 | 0.82 (0.60-1.11) | 0.191 | 0.72 (-0.52-0.98) | 0.036 | 492/595 | 1.05 (0.78-1.40) | 0.761 | 0.90 (0.66-1.22) | 0.486 |
|  |  |  |  |  |  |  |  |  |  |  |
| 6-23 | 677/831 | REF |  | REF |  | 771/937 | REF |  | REF |  |
| 24-59 | 242/295 | 1.04 (0.74-1.47) | 0.829 | 1.19 (0.84-1.70) | 0.330 | 269/326 | 1.02 (0.73-1.42) | 0.925 | 1.15 (0.82-1.62) | 0.413 |
| Yemen |  |  |  |  |  |  |  |  |  |  |
| Male | 121/201 | REF |  | REF |  | 139/215 | REF |  | REF |  |
| Female | 77/133 | 0.91 (0.58-1.42) | 0.675 | 0.90 (0.57-1.42) | 0.660 | 95/165 | 0.87 (0.56-1.33) | 0.508 | 0.83 (0.54-1.29) | 0.415 |
|  |  |  |  |  |  |  |  |  |  |  |
| 6-23 | 81/150 | REF |  | REF |  | 115/180 | REF |  | REF |  |
| 24-59 | 117/184 | 1.49 (0.96-2.31) | 0.077 | 1.55 (0.99-2.43) | 0.054 | 119/190 | 0.95 (0.62-1.45) | 0.802 | 0.96 (0.62-1.49) | 0.867 |
| South Sudan |  |  |  |  |  |  |  |  |  |  |
| Male | 329/538 | REF |  | REF |  | 561/940 | REF |  | REF |  |
| Female | 268/415 | 1.16 (0.89-1.51) | 0.279 | 1.01 (0.76-1.33) | 0.956 | 491/769 | 1.10 (0.98-1.45) | 0.078 | 1.06 (0.86-1.30) | 0.600 |
|  |  |  |  |  |  |  |  |  |  |  |
| 6-23 | 324/516 | REF |  | REF |  | 691/1119 | REF |  | REF |  |
| 24-59 | 273/437 | 0.99 (0.76-1.28) | 0.919 | 0.95 (0.71-1.25) | 0.704 | 361/590 | 0.98 (0.80-1.20) | 0.819 | 0.87 (0.69-1.09) | 0.223 |
|  |  |  |  |  |  |  |  |  |  |  |

This table represents results from 5 sets of logistic regression models; ORs represent the likelihood of recovery compared with all other outcomes for each country

*adjusted for sex, age, HAZ at baseline and WHZ at baseline,

Supplementary table 2. Multinomial analysis to assess the association between treatment outcomes and sex within TFP.

|  | Crude |  |  | Adjusted |  |  |
| --- | --- | --- | --- | --- | --- | --- |
|  |  |  |  |  |  |  |
|  | RR (95%CI) Female vs male (reference) | Standard Error | P value | RR (95%CI) Female vs male (reference) | Standard Error | P value |
| Death | REF |  |  | REF |  |  |
| Recovered | 4.21 (0.47-3769) | 4.71 | 0.199 | 2.46 (0.27-22.84) | 2.80 | 0.428 |
| Default | 3.80 (0.42-34.20) | 4.26 | 0.233 | 2.73 (0.29-25.37) | 3.11 | 0.377 |
| Transfer | 4.71 (0.52-42.60) | 5.29 | 0.168 | 3.46 (0.37-32.39) | 3.95 | 0.276 |
| Non-response | 4.31 (0.46-40.22) | 4.91 | 0.200 | 3.46 (0.36-33.34) | 4.00 | 0.282 |
|  |  |  |  |  |  |  |
| Recovered | REF |  |  |  |  |  |
| Death | 0.24 (0.03-2.13) | 0.27 | 0.199 | 0.41 (0.04-3.76) | 0.46 | 0.428 |
| Default | 0.90 (0.77-1.07) | 0.08 | 0.231 | 1.11 (0.93-1.32) | 0.10 | 0.245 |
| Transfer | 1.12 (0.88-1.42) | 0.14 | 0.352 | 1.41 (1.10-1.80) | 0.18 | 0.007 |
| Non-response | 1.02 (0.66-1.59) | 0.23 | 0.917 | 1.41 (0.89-2.22) | 0.33 | 0.145 |
|  |  |  |  |  |  |  |
| Default | REF |  |  |  |  |  |
| Recovered | 1.11 (0.94-1.30) | 0.09 | 0.231 | 0.90 (0.76-1.07) | 0.08 | 0.245 |
| Death | 0.26 (0.03-2.36) | 0.29 | 0.233 | 0.37 (0.04-3.40) | 0.42 | 0.377 |
| Transfer | 1.24 (0.95-1.62) | 0.17 | 0.117 | 1.27 (0.96-1.67) | 0.18 | 0.091 |
| Non-response | 1.13 (0.71-1.79) | 0.27 | 0.597 | 1.27 (0.79-2.03) | 0.31 | 0.325 |
|  |  |  |  |  |  |  |

Table shows the crude and adjusted odds of females reaching each outcome over the baseline outcome compared with males in TFP.
